# User Orientation Detection in Relation to Antenna Geometry in Ultra-Wideband Wireless Body Area Networks Using Deep Learning

**DOI:** 10.3390/s24072060

**Published:** 2024-03-23

**Authors:** Sebastian Urwan, Krzysztof K. Cwalina

**Affiliations:** 1 Intel Technology Poland Sp. z o.o., 80-298 Gdańsk, Poland; 2 Faculty of Electronics, Telecommunications and Informatics, Gdańsk University of Technology, 80-233 Gdańsk, Poland

**Keywords:** body area networks, deep learning, UWB, LoS, NLoS, orientation

## Abstract

In this paper, the issue of detecting a user’s position in relation to the antenna geometry in ultra-wideband (UWB) off-body wireless body area network (WBAN) communication using deep learning methods is presented. To measure the impulse response of the channel, a measurement stand consisting of EVB1000 devices and DW1000 radio modules was developed and indoor static measurement scenarios were performed. It was proven that for the binary classification of user orientation, neural networks achieved accuracy that was more than 9% higher than that for the well-known threshold method. In addition, the classification of user position angles relative to the reference node was analyzed. It was proven that, using the proposed deep learning approach and the channel impulse response, it was possible to estimate the angle of the user’s position in relation to the antenna geometry. Absolute user orientation angle errors of about 4–7° for convolutional neural networks and of about 14–15° for multilayer perceptrons were achieved in approximately 85% of the cases in both tested scenarios.

## 1. Introduction

A radio sensor network (RSN) comprises a group of wireless communication devices located in a specific area, for the purpose of obtaining information from the area and transferring it between network components. The primary purpose of radio networks is to transmit data wirelessly over distances greater than a single-wire link between their nodes. In this way, both data and short packets can be sent as utility information, based on which additional information can be obtained. This is required, for example, for synchronization or radiolocalization services.

In stadiometric navigation systems, the user’s position is determined based on the distance measured between the tracked object and a set of reference nodes with known and fixed positions. The estimation of the distance between the nodes can be made using several parameters, e.g., the Received Signal Strength Indicator (RSSI), using the relationship between the distance and the propagation attenuation of the radio signal.

The conditions in the radio link in the form of a line of direct visibility between two network nodes play a key role in the above-mentioned radiolocation applications [1]. Accurate distance measurement is a complex task due to the additive white Gaussian noise (AWGN) and multipath propagation components (MPCs) that are present in the received signal. Moreover, in non-line-of-sight (NLoS) conditions, radio signals propagate through several obstacles, as well as through the human body. In order to identify NLoS conditions, kurtosis, the variance of the estimated distance, or the power of the first component can be used [2].

The use of radiolocation systems for the estimation of coordinates in the local reference system is not the only method of obtaining information about the user’s location. Taking into account the type of radio link with the location of the node on the human body, information about the occurrence of LoS or NLoS conditions can also be used as binary information about the orientation of the user’s position.

This research focuses on the analysis of the estimated channel impulse response (CIR) in order to determine the user’s orientation in relation to the antenna geometry using a deep learning approach. The presented approach should be treated as an extension of the research described in [3,4]. Its novelty, in comparison to the current state of the art, mainly involves determining the direct angle of the user’s position and increasing the effectiveness of determining the user orientation using convolution networks and CIR estimation. In addition, the influence of the user’s orientation relative to the antenna geometry on the final efficiency of the deep learning approach is investigated.

This paper is divided into five sections. In addition to the Introduction (Section 1), in Section 2, a description of the ultra-wideband (UWB) technique as well as the use of the CIR is presented. In Section 3, an overview of the research and results presented in the scientific literature and the methods of detecting users’ orientation with the use of artificial intelligence (AI) are described. In Section 4, the obtained results concerning the efficiency of detecting users’ orientation with respect to the antenna geometry for different levels of detail of the output information (binary classification of LoS and NLoS conditions, classification of many orientation angles and determination of the angle value from the continuous range 0–360°) are presented. Section 5 is a summary of the achieved results, and it also indicates possible future work concerning the detection of a user’s orientation.

## 2. UWB Technique

UWB signals are unambiguously defined by various telecommunications organizations. The European Telecommunications Standards Institute (ETSI) defines a UWB signal as a radio signal occupying a bandwidth exceeding 50 MHz at the level of −10 dBc, i.e., −10 dB, relative to the power density at the carrier frequency [5,6]. The European Commission defines the following criterion: a radio signal is a UWB signal with a bandwidth of no less than 50 MHz [7]. The advantage of using a wide frequency band with the UWB technique is the possibility of transmission with higher bit rates, up to hundreds of Mbps. Another significant advantage of using UWB is the high time resolution, which enables distance measurements to be carried out with high accuracy and a high resolution to measure the components of the impulse response of the radio channel in nanoseconds. Nowadays, UWB techniques are commonly used in RSNs and also in wireless body area networks (WBANs) according to the IEEE 802.15.6 standardization [8].

### 2.1. UWB Devices

Various solutions and their implementations related to the UWB technique are currently available on the market. There are, for example, radio modules and evaluation devices such as the Ubisense series 7000, SpoonPhone and Decawave EVK1000. These solutions differ in terms of the device price and its intended use (working in the reference node or end node mode), the center frequency of the radio channel, the localization systems programmed in the microcontroller using time of arrival (ToA), time difference of arrival (TDoA) or angle of arrival (AoA) measurements and the resolution of the measurements taken. In [9], the effectiveness of localization was verified using UWB and commercially available Ubisense, SpoonPhone and Decawave devices. The assessment was carried out in NLoS conditions in an industrial warehouse with a variety of equipment (robots, vehicles, railways, etc.), which significantly affected the CIR. For these conditions, the higher performance of the radiolocalization system using TDoA measurements on Decawave devices compared to BeSpoon and Ubisense devices was proven.

### 2.2. UWB Applications

In the literature, the results of research on the use of UWB in RSNs supporting medical care, e.g., in detecting patient movement during rehabilitation, in sports medicine, in geriatric cases and in walk analysis, are described. In addition, UWB-based systems can be used to measure changes in the movement patterns of Alzheimer’s and Parkinson’s patients over a longer period of time. In [10], the original structure of a motion classification system using inertial sensors is described, i.e., each sensor node has a three-axis accelerometer, a two-axis gyroscope, a microcontroller and a radio module. Each node’s processing unit receives samples of the sensor readings and transmits the data to the base station. The standard deviation, peak-to-peak amplitude and root mean square (RMS) power were used as data in the system.

In addition to medical applications, the results of using such systems, for example, to locate objects in museums, where it is not necessary to determine the user’s precise location, are described. In [11], BeSpoon UM1000 devices with Bluetooth Low Energy (BLE) and UWB technology were utilized. Tests were carried out in a corridor that was 2.5 m wide and 5 m long. An important conclusion in the context of this research was that, in addition to determining the location, it is also possible to establish the user’s orientation angle in space. It was noticed that when only one reference device was used, it was not possible to determine the orientation angle as expected. However, the combined use of one reference device and the pedestrian dead-reckoning (PDR) method made it possible to estimate the orientation angle. In real-world experiments, 80% of user position angle estimates yielded an error of less than 30° for six reference devices using both the BLE and UWB techniques.

It is well known that the global positioning system (GPS), in the presence of obstacles and in the indoor environment, is characterized by low user location accuracy and, in certain cases, by the unavailability of location services. UWB-based systems, on the other hand, can mitigate the negative effects of multipath propagation and can work well for indoor locations. The benefits of each system, i.e., GPS for location in outdoor environments and UWB for location in indoor environments, were adopted to develop a hybrid radiolocation system [12]. In the study, the authors confirmed that the proposed GPS-UWB system did not reduce the time of location services’ availability due to the additional measurements resulting from the use of the UWB technique, and it reduced the sensitivity to the initial determination of the user’s position.

In [13], the use of UWB in radars for the control of car traffic to prevent collisions while driving and parking is presented. The study also mentions its application in security systems to detect unauthorized intrusions in a guarded area, to locate people buried under construction obstacles or snow slides by their movements or heartbeats and in the remote measurement of the heart rate, respiratory activity and other vital parameters of patients. The above-mentioned tasks, in the context of medical care, are implementations of the strategy of active and assisted living (AAL) [14], which seeks to ensure and promote the independent living of the elderly in their homes.

Most of the solutions presented in this section are based on the use of UWB for location services based on the measurement of the propagation times of ToA radio signals and on the detection of NLoS conditions. The authors of [15] proved the significant effect of the orientation of transceiver antennas relative to each other, as well as their polarization, on the detection of human breath. In the literature, solutions for the detection and distinguishing of people from other objects, e.g., behind a wall, using a UWB radar [16] and the two-dimensional fast Fourier transform (FFT) [17], are also proposed. In addition, the detection of direct visibility conditions with a UWB radio interface in node-to-node communication was presented in [18,19,20], and, in [21], the authors investigated several other types of obstacles, such as wood, steel and the human body.

## 3. Method for Detection of Localization of Users

In the current state of the art, issues related to the location of devices in Internet of Things (IoT) networks are often discussed, because determining the exact location may be, among others, the basis for the correct interpretation of the transmitted user’s information [22]. As already mentioned, the GPS system is characterized by significantly lower efficiency in the indoor environment and therefore other positioning methods are used [23,24]. Moreover, on the basis of the research described in the literature, the negative influence of NLoS propagation conditions on the accuracy in estimating the distance using the ToA method can be determined. This significantly influences the accuracy in determining the positions of modules using the circular method [25]. The currently available methods for the identification and minimization of the positioning error for NLoS conditions in UWB are divided into several categories for dense environments. The methods used to identify NLoS are as follows [26]:

- the range estimation method—uses estimated distance variance measurements and time series measurements to compare with a certain threshold;
- the radio channel parameter method—identifies the channel by analyzing the parameters of the received radio signal, i.e., the received signal power, the power of the first component of multipath propagation, the average delay and the amplitude and signal to noise ratio (SNR) in the receiver;
- the building maps method—identifies the channel by observing the previous position of the mobile user or the environment.

Therefore, there are techniques to minimize the impact of NLoS conditions [26]:

1. the direct path detection method—finds the direct path of the received signal reaching the receiver via various paths; the direct path usually provides more accurate information as compared to the multipath propagation component of the received signal with the highest power;
2. statistical methods using least squares, weighted least squares, Taylor series, the linear programming approach and filtering.

The methods of extracting the parameters of the radio channel are particularly important in detecting the user’s position (and orientation) in relation to the antenna geometry in the UWB radio link. The radio channel parameters obtained on the basis of the stored instantaneous CIR and the averaged CIRs are commonly used nowadays to identify LoS and NLoS conditions. The main problem in radiolocalization systems is the influence of the harsh propagation environment—in particular, propagation under NLoS conditions. To improve the location accuracy under such conditions, NLoS condition identification algorithms are first used, followed by algorithms to minimize the impact of errors caused by these conditions. Examples of such algorithms are the methods based on the received signal power envelope statistics [27].

### 3.1. Measurement Setup

In this research, a Decawave EVK1000 kit consisting of two EVB1000 evaluation devices was used (Decawave, Dublin, Ireland). For the purposes of this work, one EVB1000 device was used as a reference node powered by a computer with a USB port and the other one as a wearable node powered by an external battery and placed on the human body. The microstrip antennas supplied with the EVK1000 set were used. The configuration of the EVK1000 was set as mode 3, i.e., channel no. 2, with a carrier frequency of 3993.6 MHz, a bandwidth of 499.2 MHz, a transmission power spectral density of −41.3 dBm/MHz and a pulse repetition frequency (PRF) of 64 MHz [28,29,30,31,32,33].

### 3.2. Measurement Scenarios

**Figure 1:**
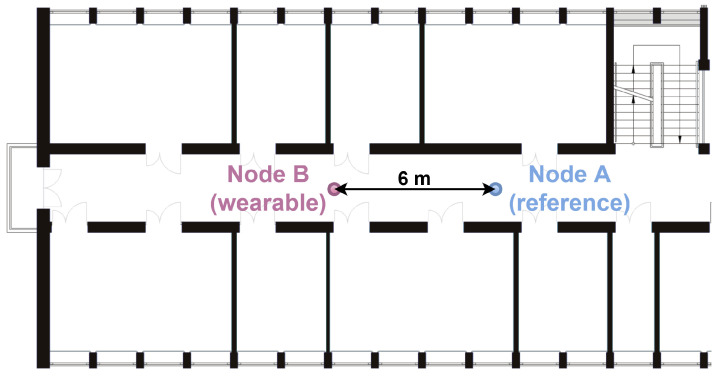
Two-dimensional plan of the corridor inside the building (S1 scenario).

**Figure 2:**
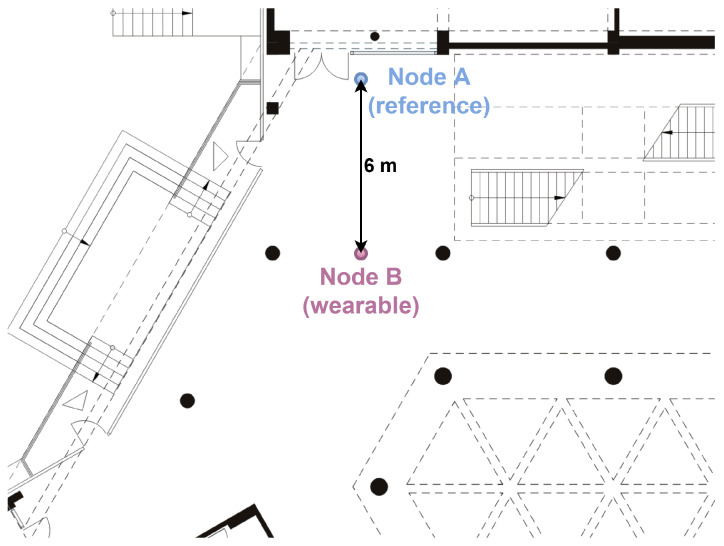
Two-dimensional plan of the hall inside the building (S2 scenario).

In order to verify the effectiveness of the methods used to detect the user’s orientation in different propagation conditions, several measurement scenarios for off-body communication were developed. Currently, for the discussed type of UWB radio transmission, indoor scenarios, including laboratory rooms, office spaces [34], corridors [11] and lobbies [4], are used. It is worth emphasizing that, in the literature, the results of research on NLoS condition classification for dynamic scenarios [4,35] are also presented. Due to the fact that the designed off-body WBAN sensor networks are used in indoor environments, where the impact of multipath propagation cannot be neglected, it was decided that the measurement campaigns would be carried out similarly to [4], in indoor environments in the 4th-floor corridor of the PG WETI A building and in the main hall on the ground floor of the PG WETI B building. These locations are denoted as S1 and S2, respectively. In Figure 1 and Figure 2, fragments of the plans of the above-mentioned buildings are shown.

A man weighing 71 kg and 187 cm tall participated in the measurements. The wearable node was placed on his belt at a height of 110 cm, and the reference node was placed on a tripod stand, also at the height of 110 cm.

It should be noted that, during the subsequent analysis, it will not be possible to directly compare the results described in the current state of the art. Despite the similar dimensions of the rooms, corridors and halls used in [4,36] and in this research, the main problem is the verification of the entire environment’s impact on the radio waves’ propagation, e.g., elements of the room furniture and furnishings, the wall thickness, the building materials used, the presence of moving people inside or the presence of other radio transmissions in a similar frequency range. Therefore, the obvious limitation of such an approach is to unequivocally determine the most effective of the tested methods based on the current state of the art, because the environments in which the experiments are carried out directly affect the obtained results regarding the classification accuracy.

Furthermore, the effectiveness of WBANs, particularly UWB systems, is significantly influenced by the human body. In [4], it was proven that the characteristics of the human body may, in certain cases, be far more influential than the propagation environment on the classification efficiency. In the study, the authors noted a significant reduction in the classification efficiency (from 7.4% to 9.5%) obtained by deep neural networks for people with more fat and muscle tissue. At this stage in the research, measurement scenarios were applied for a single person to verify the possibility of using the deep learning approach for orientation detection in relation to the antenna geometry. This should thus be considered an extension of the previous research, offering opportunities for further investigation, e.g., with multiple environments and multiple people.

**Figure 3:**
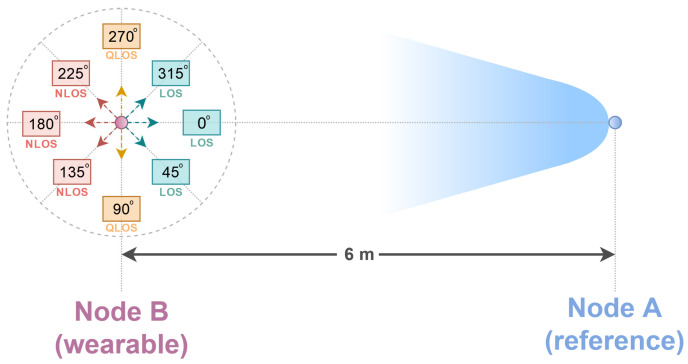
Angles of the user’s orientation relative to the reference node.

Two measurement campaigns were carried out, during which the impulse responses of the radio channel and the power of the received radio signals were obtained. In Figure 3, a diagram of the measurement campaign is shown.

For each of the eight angles marked in Figure 3, i.e., angles 0° to 315°, the measurements were conducted for a period of 10 min. During the measurement for a single angle, the person with the wearable node fixed to his belt stood at a certain angle relative to the reference node (Figure 3). It should be noted that the orientation angle of the human relative to the reference node was equal to the angle of the orientation of the wearable node relative to the reference node. Applying division into a larger number of angles in the measurement campaign makes it possible to test the methods’ effectiveness not only in the binary classification of LoS and NLoS conditions, as was previously done [4], but also for the more complex task of the multiclass classification and regression of the user’s position in relation to the antenna geometry.

In order to determine the time resolution of the obtained measurements with the use of the developed stand, a test was carried out in which the impulse response of the radio channel was measured for 305 s. As a result, 2461 impulse responses were stored. The time resolution for testing in the DecaRanging program was set to 50 ms. Taking into account the time of the measurement and the final number of stored CIRs, it was found that the real time difference between the data records in the computer memory was about 124 ms. This value is close to the value of the impulse response saving period of 133 ms, which was used in [37].

During a 10-min measurement for a single angle, an average of 5431 CIRs were recorded. In addition, filtering of the initial and final measurement data was carried out in order to equalize the size of the measurement datasets. Ultimately, the measurement dataset for a single angle consisted of 4800 impulse responses, so the total measurement dataset for one measurement scenario consisted of 38,400 impulse responses, with each one consisting of 1016 samples. The resolution of the impulse responses was set to 150 ms and the resolution of the impulse response samples was 1 ns. This size was similar to the sizes of the datasets used in the literature, e.g., in [38,39], which used a dataset containing 42,000 impulse responses.

### 3.3. Threshold Methods

In the UWB technique, the issues of the localization and classification of LoS and NLoS conditions are widely described and analyzed. Among the classic methods, the threshold method can be distinguished. It involves the analysis of parameters calculated on the basis of the impulse response of the channel, e.g., kurtosis, mean excess delay or RMS delay spread [40], or the analysis of the power values of the received signals in the receiving part, i.e., the total power $P_{rx}$ and the power of the first component of the multipath propagation $P_{fp}$ of the received signal [41].

Therefore, the measurements of the received signals taken for the purposes of this research could be directly used to detect LoS and NLoS conditions between the reference module and the wearable module placed on the human body. The detection of NLoS conditions can be achieved by comparing the amplitude of the direct component with the largest received multipath component [42]. The thresholding methods applied include extracting significant features from the channel measurements, which allows for the calculation of the difference between the feature values. Then, based on the comparison of the obtained difference and the determined threshold value, the condition classification is performed. In this study, the difference between $P_{rx}$ and $P_{fp}$ was adopted.

To determine NLoS conditions, the authors of [41] used the method of calculating the power performance index $P_{pi}$ (difference between $P_{rx}$ and $P_{fp}$) and the RMS delay spread, where these parameters were determined on the basis of the impulse response. The results showed that the use of both parameters for the classification of NLoS conditions in the indoor environment described by the authors was characterized by high classification efficiency of 96.3% and 96.69%, respectively. For the above methods, the effectiveness parameters were also determined, i.e., false negative (3.09% and 2.54%, respectively) and false positive (0.61% and 0.77%, respectively).

In [43], an NLoS identification algorithm using the total signal power, the maximum amplitude, the normalized power of the first component of multipath propagation, the SNR, the rise time, kurtosis and the mean excess delay parameter was proposed. The authors separated the first signal path and the multipath signal from the channel data and compared them for NLoS identification, achieving 93.9% accuracy in the classification of NLoS conditions and 92% accuracy for LoS conditions. In addition, NLoS detection solutions based on a particle filter are presented in [44]. Classical algorithms are simple enough to function in UWB devices. However, it should be emphasized that most algorithms use fixed environment-dependent threshold values to classify NLoS conditions [34].

In [45], the results of research carried out in a laboratory environment using DW1000 radio modules with the classic threshold method and machine learning methods, including a multilayer perceptron (MLP) and binary decision tree (BDT), for the detection of LoS and NLoS conditions, are described. According to the Decawave documentation, a threshold of 10 dB was assumed as the difference between the value of the total power of the received signal and the value of the power of the first component of multipath propagation. Machine learning methods were adopted as the reference level for the results and as the input characteristics, based on which the models were trained. The authors chose three parameters: the distance measurement, the amplitude of the first component of the multipath propagation and the standard deviation of the noise in the recorded impulse response of the radio channel. A total of 3152 samples (1531 LoS and 1621 NLoS) were used to train the MLP and BDT models. The results for the stationary scenario proved that both models provided the same accuracy (98%) as the classical threshold method with a threshold of 10 dB. However, in the dynamic scenario, the accuracy of the threshold method decreased by 10%. Changing the threshold value to 8 dB improved the classification accuracy to 91%. The authors observed that the machine learning models were more efficient than the threshold method, with MLP efficiency of 92% and BDT efficiency of 94%.

To summarize, specific attention should be paid to the limitations of the classic threshold method, with the key problem of selecting the correct threshold value, which may be different for each measurement scenario. Another limitation of the above method is its exclusive application in the binary classification of LoS and NLoS conditions.

### 3.4. Deep Learning Methods

Machine learning methods are included in the concept of artificial intelligence. In general, ongoing research related to the field of deep learning is aimed at adapting a network of information processing units to solve non-linear problems by following certain rules or using a defined set of data.

In the context of the discussed issue of LoS and NLoS classification, some approaches based on artificial intelligence methods have already been proposed, e.g., convolutional networks and a multilayer perceptron as an example of a classical, feedforward network. In the studies described in [34], the approach of machine learning was used and the impact of training neural networks was checked to enable its use in environments characterized by different propagation properties. In addition, in recent years, due to technological progress, Internet of Things (IoT) networks have found many applications, especially as sensor networks with location services in indoor environments. As mentioned earlier, there are hardware and software solutions that offer greater accuracy in the location of users inside buildings. However, solutions involving more parameters, increasing the accuracy of user location, are constantly being developed. An example is the classification of LoS and NLoS conditions, which may directly affect the functioning and efficiency of the entire localization system.

In [45], the study of the impact of NLoS detection as the first stage of the entire system’s operation was presented. The authors proposed the Naive Bayesian (NB) algorithm for the classification of NLoS conditions in order to improve the accuracy of the localization system, using time of arrival (ToA) measurements with the UWB technique. The results showed that the error between the actual distance and the measured distance increased with the increase in the distance between the end nodes and the reference node. The efficiency was determined based on the area under the curve, representing the relationship between the true positive and false positive rates, and it was approximately 87%. The authors determined that this method can contribute to increasing the accuracy of positioning in NLoS conditions.

The issue of detecting NLoS conditions using the impulse response of the radio channel is also solved with the use of convolutional neural networks [46]. This type of topology consists of three types of layers, convolutional, sampling and dense layers, used in a multilayer perceptron. In addition to the classic convolutional network, other network variants are used in the literature, such as a residual network (ResNet) or encoders [46].

Taking into consideration the previously mentioned results and conclusions, as well as the advantages of deep learning approaches in terms of direct visibility condition detection over other methods [3,4], it was decided that deep learning methods would be analyzed in the subsequent research.

#### 3.4.1. Training, Validation and Testing of Neural Networks

Neural networks contain different layers of interconnected nodes (neurons) that transmit signals to the activation functions, which are usually non-linear in order to increase the complexity of the network model. In general, the supervised training of a neural network proceeds in the following steps: initializing the initial weights of the node connections, obtaining the results from the neural network, calculating the error based on the obtained results and correcting the results by adjusting the weights to reduce the error value. These steps are repeated for all training data.

In neural networks, the features of one input vector are assigned as the input layer. The input values are propagated through the nodes of successive hidden layers and multiplied by the weight matrix. A loss function $L(y^{\prime },y)$ is also defined and it depends on the resultant value $y^{\prime }$ calculated by the network and the expected value of *y*. After the progressive propagation step, backpropagation takes place, using gradient algorithms to optimize the model weights. Optimization algorithms such as RMSProp or ADAM [47] start the optimization process by calculating the gradient in the output layer and act backwards towards the input layer. The result of the gradient calculation is an indicator of the changes to the weight values, which reduce the resulting network error. The gradient for the output layer, which will be propagated to the last hidden layer, is described as

(1) $$g\leftarrow \nabla_{y^{\prime }}L\left(y^{\prime },y\right).$$

The training stage of the neural network model is carried out in accordance with the assumptions made by the designer, with one training iteration being the calculation of the cost function and backpropagation of the error for the entire training set, i.e., for all training subsets [47]. The simplest solution in this case is to assume a fixed number of iterations. However, this provides less control over the whole training process. An exemplary value of 20 iterations was selected in [39]. The use of a constant value may result in the overfitting (overtraining) or misfitting of the network model. Overfitting occurs when the difference between the training error and the error in the test data is large, which means that the node connection weights in the network model are too fit to solve the problem in the training dataset. Underfitting, on the other hand, denotes the premature termination of the training process, which results in a large training error in the network model.

Depending on the problem to be solved by the network, the type of network, the selected hyperparameters and the number of iterations of the training process may vary. For this purpose, the method of adaptively stopping the model training after certain conditions were met was implemented. It is worth mentioning the use of the so-called set of validation data that are not directly used to train the network model but allow one to check for misfitting or overfitting, e.g., at the end of each iteration of the [47] training process. By utilizing the measurement data from the same experimental sample for the training and testing phase, it will be possible to determine the estimated generalization error after the model learning phase is completed. It is important that, when splitting the dataset, none of the measurement examples appear simultaneously in the training, validation and test sets.

The last stage of each iteration of the network model training is to verify the effectiveness of the current state of the model, i.e., all the connection weights and intercepts for the set of validation data unknown to the network model. Calculating the fit error of the model to the validation dataset allows one to check, in each iteration of the training, the condition that determines misfitting or overtraining. This condition applies when exceeding the maximum value of the iteration counter for which the specified criteria have not been met. If, in a given training iteration for the validation process,

- the value of the loss function is greater than or equal to the previous smallest value of the loss function, or
- the absolute difference between the value of the loss function and the value of the loss function from the previous iteration is less than the set threshold,

the counter value is increased by one and exceeding the counter value of 10 results in the end of the training phase. In addition, the maximum training iteration threshold of 400 was declared to prevent an overly long training process. A detailed and in-depth mathematical description of the used deep learning approach can be found in [47].

The implementation of deep neural networks was performed using the Python 3.9 programming language and the PyTorch 1.9 library. The models were trained and tested on a computing machine with a Ryzen 5 3600 processor (6 cores clocked at 3.59 GHz), 32 GB RAM, a GTX 1660 Super (GPU) and 512 GB SSD in the ProtoLab laboratory of the Gdańsk University of Technology.

#### 3.4.2. Input and Output Data

The scope of the research using deep learning methods in the classification of NLoS conditions is being constantly expanded. It also involves the optimization issue by providing various measurements as the input of a neural network or by testing various types of neural networks and their architectures. However, the current state of the art emphasizes the significant impact of using the entire impulse response of the channel as the input data of neural networks on the computational complexity of data processing, thus extending the training time of the network models. Therefore, in [39], an original approach, involving the pre-processing of the channel impulse response in the form of CIR subsampling, was proposed. The results of this classification showed that the use of a reduced number of radio channel impulse response features results in similar effectiveness to that of a full response. However, as expected, a fourfold decrease in the training time was observed. The authors emphasized that the accuracy results were obtained for the dataset created in specific propagation conditions, and it is not possible to directly compare these results to the studies described in the literature.

Typical proportions of the data subset used to train a neural network are 80% of the data for training and 20% for validation [47]. Researchers indicate similar divisions of datasets. In [39], the whole dataset was split as follows: 60% for the training set, 20% for the validation set and 20% for the test set. Taking into account the use of cross-validation, in the present research, 80% of the entire dataset was used for the training and validation phases, and 20% for the network testing phase. On the other hand, the dataset for the training stage was divided into 80% for the training process and 20% for validation. Other parameters important for the training of neural networks are the sizes of the training, validation and test datasets. For further research related to deep learning, training sets were created for each measurement scenario. They consisted of 4800 measured impulse responses of the radio channel and of the received signal power values for each of the eight angles (Figure 3), which yielded a total of 38,400 instantaneous CIRs.

Having obtained the information about the applied division and size of the individual datasets, one should consider the format and meaning of the data as a single input vector, which may directly translate into the achieved values of the network model’s efficiency. As described previously, the available data are the measured impulse responses of the radio channel and the received signal power values corresponding to these responses. To analyze the tasks of neural networks related to the classification of LoS and NLoS conditions, two data approaches were used in the input layer: normalized complex values of the impulse response and normalized power values of the repelled signals. The purpose of this division was to check the impact of the input data type on the efficiency of a given neural network.

During further research related to the classification of the eight user orientation angles and to the regression problem for the user’s orientation angle, only the normalized values of the impulse response samples were used. The real and imaginary values of the radio channel impulse response samples were normalized and corresponded to the minimum and maximum values that could be written to 16 bits of a signed integer. The values obtained from the measurements were therefore normalized to the range of −1 to 1 [48]. The power values of the received signals were normalized to the range of 0 to 1 [48] using $P_{min}=-123.5\;$[dBm]. This was the minimum power value that was measured. It was additionally reduced by 3 dB and $P_{max}=-74.4\;$[dBm], which was the value of the received signal power specified in the documentation, taking into account only the frequency of the radio wave and the signal attenuation resulting from the distance between the transmitter and the receiver [31].

The output layer is characterized by a different number of nodes depending on the issue being solved. For the binary classification of LoS and NLoS conditions, there is one output node. While specifying one of the eight classes of angles, eight nodes were used in the output layer, as in multiclass classification approaches [49]. In the case of orientation angle regression, there is one node at the output of the neural network and it is the value of the estimated angle in the range of 0 to 1, which is scaled to the range of 0° to 360°.

#### 3.4.3. Hyperparameter Tuning

The network parameters, the so-called hyperparameters, define the architectures of neural networks and directly affect the training process (e.g., its optimization algorithm) and the final network’s effectiveness as well. Depending on the type of task to be solved by the network and the design assumptions, such as the network efficiency, the hyperparameter values should be selected considering the accuracy of the results to be achieved and the reduction of the computational complexity. It needs to be pointed out here that, during network training, information about the hyperparameters’ optimal values cannot be obtained. In the current state of the art, it is the empirical values based on the conducted experiments that are usually given. As a result, it is not possible to unambiguously determine the quasioptimal values of the hyperparameters in advance.

To solve the above problem, algorithms commonly used in the literature (e.g., grid search, random search or genetic algorithms [50]) that search multidimensional spaces are used. The main problem with algorithms of this type is their considerable time complexity while searching many hyperparameters, but, on the other hand, they allow for the examination of a wide range of hyperparameter spaces and the determination of the occurrence of regions of values for which the neural networks are the most effective [47].

In order to determine the architecture of a neural network defined by the depth and width, it was decided to implement a grid search algorithm in this research. A grid search is a brute force method because, for each number of hidden layers, each number of nodes defined in the set of values is checked. Hyperparameter values obtained in this way for all the measurement scenarios were used for basic unidirectional networks, as well as in some parts of the dense network in the convolutional network. In order to search a larger range of values of the examined hyperparameters, it was assumed that the set for the hidden layers would be defined as a set in the range 1, 2, 3, 4, 5, and the set for the nodes in the range 32, 64, 128, 256. In addition, for the remaining hyperparameters, valid for multilayer perceptrons and convolutional networks, the following fixed values were selected based on the literature review:

- Learning rate: 0.0001;
- Training batch size: 64;
- Number of cross-validation iterations: 5;
- Optimization algorithm: Adam;
- Activation function: ReLU;
- Depth of the convolution part: 1;
- Number of filters: 10;
- Convolution kernel size: 4;
- Reduction layer kernel size: 2;
- Kernel shift of the reduction layer: 2.

In the case of convolutional networks, there are additional hyperparameters related to the convolutional layers, namely the depth, the dimensions of the filters and the output channels. All the hyperparameter values used in the study were determined on the basis of a review of the current research, i.e., [34,39,47,48,49,50].

### 3.5. Performance Metrics

Performance metrics are used to determine the effectiveness of the classification methods used for various problems. However, in some applications, the percentage accuracy of these methods may not be sufficient information because one type of error may be more influential than another.

It may thus be concluded that by using additional performance metrics for the classification methods, it will be possible to obtain additional information during the binary detection of the user’s orientation relative to the geometry of the antennas. The values of true positive, false positive, false negative and true negative were used to calculate additional metrics such as precision and sensitivity, which are defined as

(2) $$trp=\frac{tp}{tp+fp},$$

(3) $$ppv=\frac{tp}{tp+fn},$$

where trp is the true positive rate (precision), ppv is the positive predictive value (sensitivity), tp is the true positive rate, fp is the false positive rate, and fn is the false negative rate.

Additional research related to the problem of classifying a larger number of classes corresponding to the values of eight angles from the discrete range 0–360° and determining the angle value from the continuous range 0–360° was also carried out. It was performed using artificial intelligence methods due to the limitations of the classical threshold method. In order to determine the effectiveness of the trained classification models, the accuracy metric expressed as a percentage was used, and, for the training stage of the models solving the regression problem, the root mean square error (*RMSE*) was used. It is defined as

(4) $$RMSE=\sqrt{\frac{1}{n-1}\cdot \sum_{i=1}^{n}{|\theta_{i}-\theta_{i}^{\prime }|}^{2}},$$

where *n* is the size of the dataset, $\theta_{i}$ is the *i*-th real value of the angle in degrees, and $\theta_{i}^{\prime }$ is the *i*-th value of the user’s position angle estimated by the neural network model and is expressed in degrees.

## 4. Experimental Results

The prepared measurement setup and measurement scenarios were used to carry out the measurement campaign. On this basis, it was possible to process all the data for the statistical analysis and to create the datasets required for the classical threshold method and for the artificial intelligence methods. The measurement dataset used for one measurement scenario consisted of 38,400 impulse responses, with each impulse response consisting of 1016 samples.

### 4.1. Measurement Campaign Results

**Figure 4:**
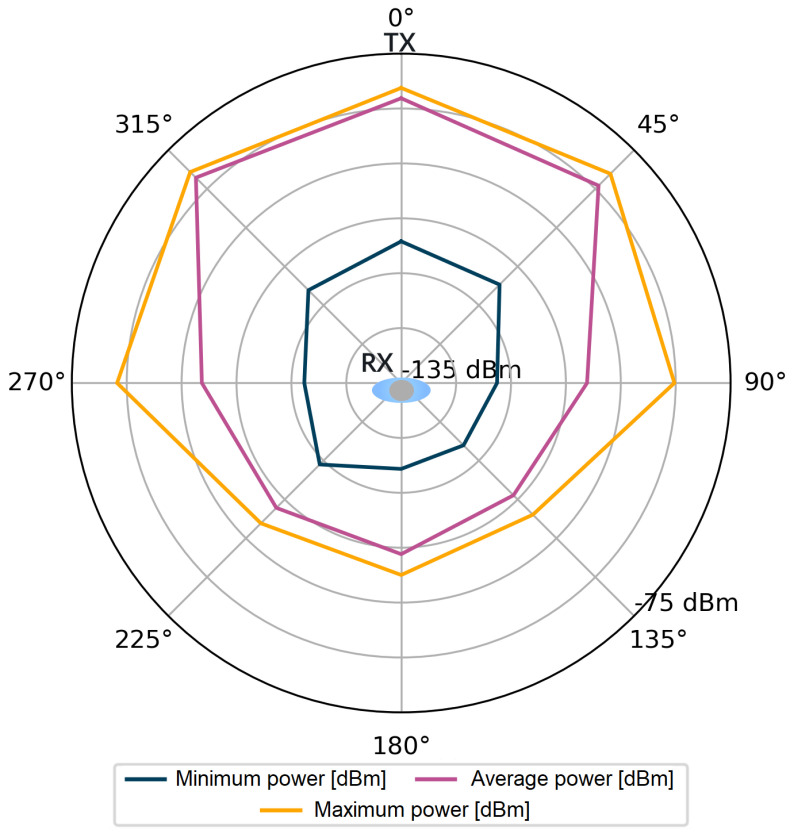
Power of the first component, scenario S1.

**Figure 5:**
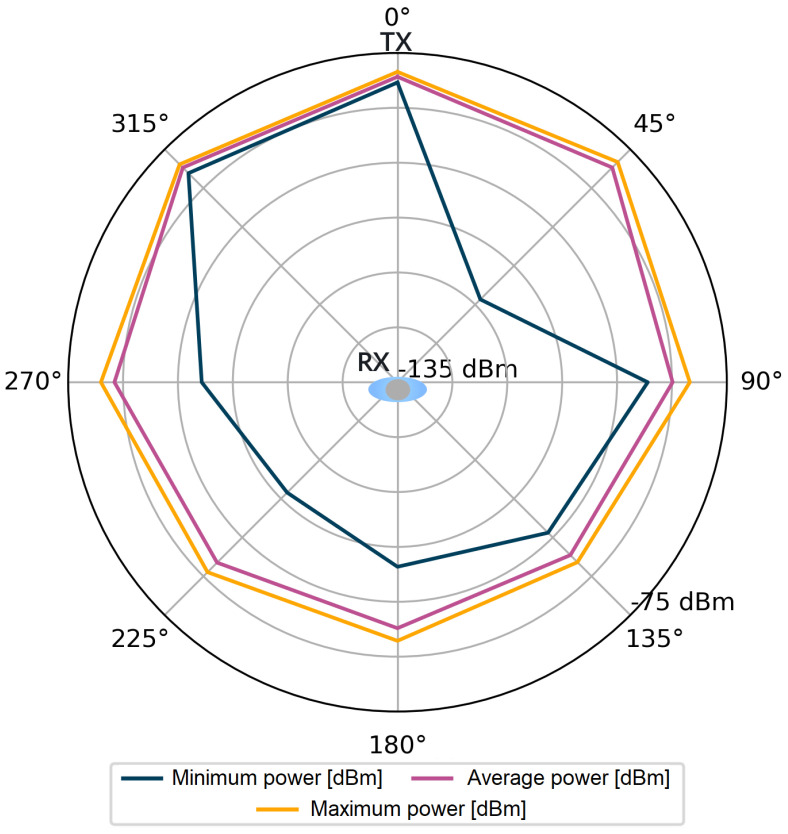
Total power of the received signal, scenario S1.

**Figure 6:**
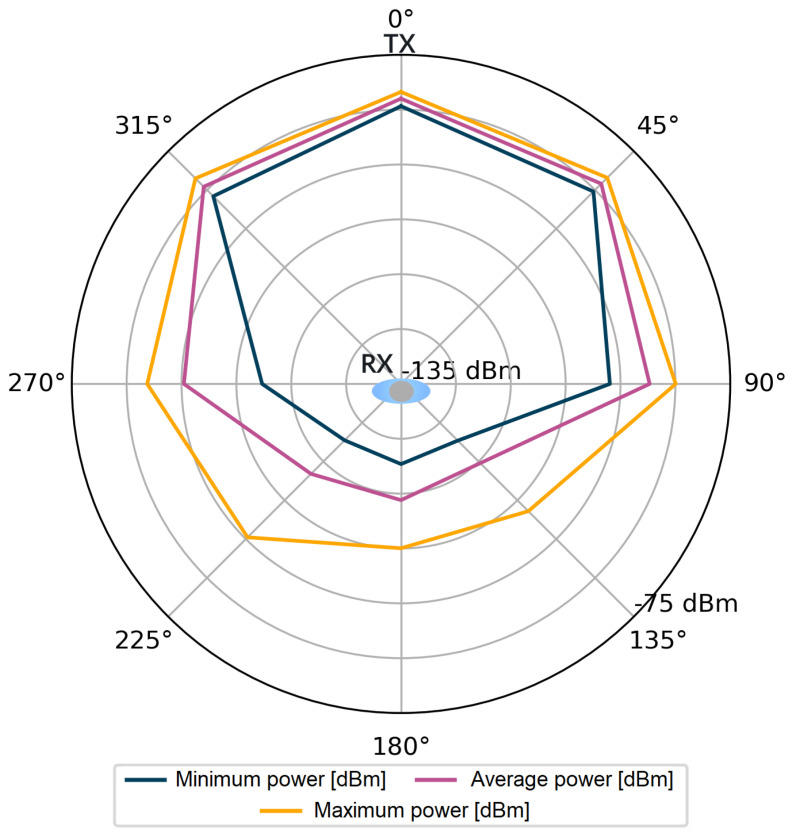
Power of the first component, scenario S2.

**Figure 7:**
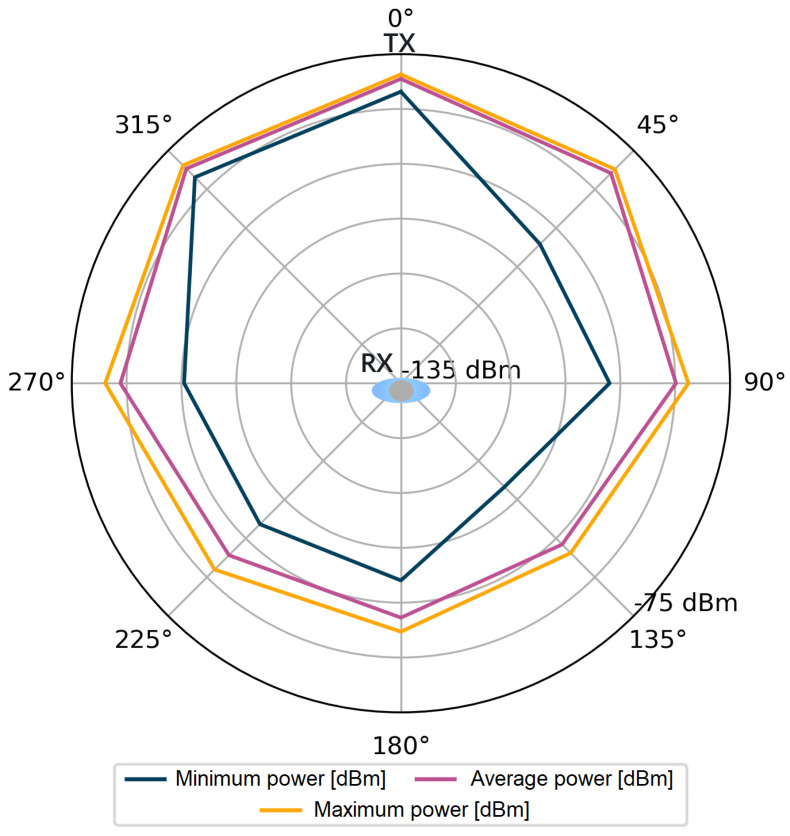
Total power of the received signal, scenario S2.

For each stored impulse response consisting of 1016 complex values, additional information was also available, such as the value of the total signal power of the CIR and the power of the first component. However, it should be kept in mind that obtaining these values in the registers of the DW1000 module is not always feasible. The number of incorrect power values for all angles in the considered measurement scenarios were counted in sets of 4800 power values for each angle. Incorrectly saved power values comprised only 0.83‰ of the measurement dataset per single angle. The stored data were used to visualize the signal power distribution for the tested measurement scenarios, which is shown in Figure 4, Figure 5, Figure 6 and Figure 7.

Following the assumptions, in Figure 4 and Figure 6, lower power values are noticeable for the first detected component. Especially for the angles in the range of 135–225°, the average power values $P_{fp}$ are lower than $P_{rx}$ by 15 dB for the S1 scenario and by over 20 dB for the S2 scenario. The average values of the total power presented in Figure 5 and Figure 7 are close to the maximum values for each angle of rotation—the difference between the maximum and average power is up to 3 dB. On the basis of the given distributions, it was noticed that, for the angles with the conditions of direct visibility between the reference and the proximal node, $P_{fp}$ was up to 5 dB lower in relation to the total signal power. In addition, comparing the results obtained for both scenarios, a significant reduction in $P_{fp}$ to −115 dBm was noted in the S2 scenario for the angle of 180°. This could be due to the characteristics of the propagation environment (the hall), which affected the distribution of the multipath propagation components.

### 4.2. Accuracy of LoS and NLoS Condition Classification for Threshold Method

The implemented classic threshold method based on two parameters of the received signal, i.e., $P_{rx}$ and $P_{fp}$, was verified on the basis of the measurement data from the S1 and S2 scenarios.

#### 4.2.1. Threshold Method Proposed by Decawave

At first, the method using the parameters compliant with the Decawave [33] approach was tested. According to the documentation, the threshold value of the difference between $P_{rx}$ and $P_{fp}$ of up to 6 dB constitutes LoS conditions, and a difference greater than 10 dB constitutes NLoS conditions. In addition, an important issue related to labeling the measurement data for the angles of 90° and 270° has been investigated here. In Table 1, the accuracy, sensitivity and precision results of the threshold method proposed by Decawave for various scenarios are presented.

Based on the obtained results, it can be seen that the impacts of classifying the angles of 90° and 270° as LoS or NLoS on the precision and sensitivity are similar in both scenarios. Including the angles of 90° and 270° as NLoS reduces the sensitivity by 15–20% compared to classifying these angles as LoS. However, the accuracy value increases by 20–25% for both scenarios. This proves that, for the angles of 90° and 270°, the share of false positive values increases and the share of false negative values decreases. The overall accuracy of the threshold method depends on the selected measurement scenario, as well as the angle division used for NLoS conditions, and ranges from 85% to approximately 90% in all the experiments performed. The accuracy difference between the measurement scenarios is smaller when classifying 90° and 270° angles as NLoS conditions and amounts to 1.66%, for which reason all further studies adopted this division.

#### 4.2.2. Effect of Threshold Changes on Classification Accuracy

**Figure 8:**
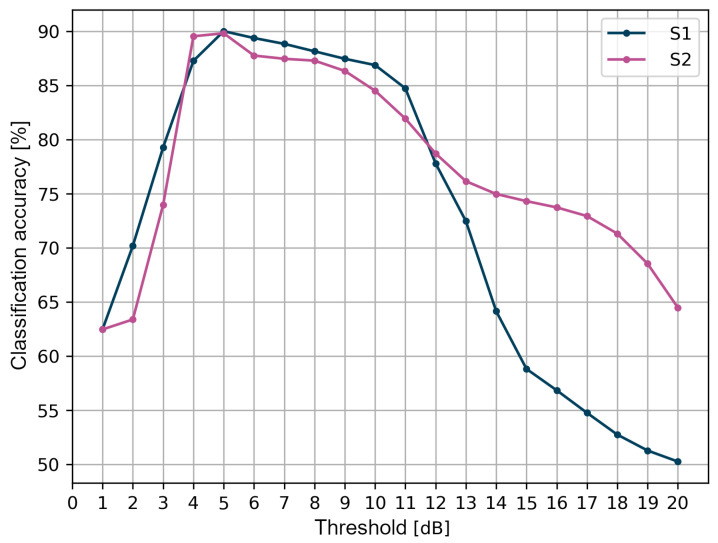
Classification accuracy of the threshold method.

Another part of the threshold method research was aimed at verifying the effect of the threshold value on the performance metrics. It should be noted that the threshold is considered here as a single value that differs for LoS and NLoS conditions and not a double value, as in the original method described in [33]. In Figure 8, the graphs of the accuracy dependent on the threshold value for the tested measurement scenarios are presented.

The highest accuracy of the binary classification of LoS and NLoS conditions, amounting to about 90% for both scenarios, was achieved for a 5 dB threshold. For values less than 4 dB, the accuracy does not exceed 80%. Up to 11 dB, for both scenarios, the accuracy value is not less than 80%. For higher threshold values, however, there is a significant reduction in accuracy, especially for the S1 scenario. An important conclusion is that, depending on the measurement scenario, i.e., the location of the measurements and other environmental factors such as propagation attenuation, the optimal threshold value may be different. The accuracy of the classification achieved is lower in these scenarios than in those presented in the literature [41], which is about 96%.

During the analysis of the obtained data, it was noticed that, for both scenarios, S1 and S2, there was a similar increase in the sensitivity value, from 0% to nearly 100% for the range of threshold values from 1 to 6 dB. For values greater than 6 dB, saturation was observed, and, for the S1 scenario, the sensitivity was 97.5%, whereas, for the S2 scenario, it was 100%. According to the definition of the confusion matrix, sensitivity determines the percentage of measurement data correctly classified as LoS out of the set of all real data marked as LoS, i.e., correctly true and falsely negative. Accordingly, for a threshold value below the optimal value set at 5 dB, even a small change in the value will result in a significant increase in the proportion of data correctly classified as LoS. Above the optimal value, the share of the values classified by the threshold method as false negative decreases significantly.

The precision results of the threshold method require a separate discussion. Between the values of 1 and 2 dB, there is a sudden increase in precision from 60% to over 80%. By definition, precision is the percentage of measurement data correctly classified as LoS out of the set of all responses of the threshold algorithm classified as LoS, i.e., correctly and falsely classified as LoS. For threshold values greater than 2 dB for the S1 scenario and greater than 4 dB for the S2 scenario, the precision decreases. This is due to an increase in false positive classifications in the dataset that should be classified as NLoS conditions.

Regarding the accuracy of binary classification, it was also possible to check the influence of each of the eight tested angles on the final accuracy of the threshold algorithm. For both scenarios, 100% classification accuracy was observed for the angles in the range of 135–225°, for which the line of direct visibility between the transmitting and receiving modules was obscured by the human body. In addition, for both scenarios, for the angles within the line of sight range, i.e., {0°, 45°, 315°}, the classification accuracy exceeded 90 %. For the S2 scenario, the exceptions were the angles of 90° and 315°, for which the accuracy was 40% and 80%, respectively. It was also noted that, for S1, for the angles of 90° and 270°, the classification accuracy was 60%, which contributed to the greatest extent to reducing the overall accuracy of the threshold method for this scenario. This is due to the fact that the applied threshold of 5 dB is inappropriate when the antenna of the wearable module is directed perpendicularly to the antenna of the reference module. In this case, it means that the difference between the received signal power and the first component oscillates around 5 dB.

### 4.3. Accuracy of Classification of NLoS Conditions with Use of Deep Learning Methods

In this section, the effectiveness of artificial intelligence methods is compared with the results of applying the classical threshold method. Potential applications of neural networks to classification and regression problems related to the detection of the user’s orientation relative to the antenna geometry, which are not possible with the use of a threshold algorithm, will also be discussed.

#### 4.3.1. Results of Selecting Hyperparameters by Grid Search

The first stage of the research related to neural networks was to check their efficiency in the binary classification of LoS and NLoS conditions. As mentioned before, the hyperparameters defining the architecture of the neural network, i.e., its depth (the number of hidden layers) and size (the number of nodes in each hidden layer), were evaluated.

Regardless of the measurement scenario and the architecture of the neural network, the average accuracy for the five models in all the tested cases exceeds 99.4%. However, determining which of the studied architectures is the most effective is problematic as the largest difference between the architectures is only 0.4%. In general, as the number of hidden layers and nodes increases, the classification accuracy increases. It is also worth emphasizing that increasing the number of dense layers in convolutional networks does not influence the efficiency of the network models. Similar dense layer architectures can be used for both multilayer perceptrons and convolutional networks. The most efficient architecture is not obvious because, in addition to the highest average classification accuracy among all five models of the same architecture, the training time of the neural network should also be taken into account. In the case of a convolutional network with 1 hidden layer and 32 nodes, the average training time for five models is 41 s; for the architecture with 4 hidden layers and 256 nodes, it is 51 s. For the multilayer perceptron case, identical architectures have an average training time of 15 s.

In further research, to check the effectiveness of neural networks for other problems related to the detection of the position orientation, the following architecture was used: 4 hidden layers and 256 nodes. It had the highest average accuracy of 99.81% for all five models of the same architecture.

It can be concluded that the type of network and the values of the examined hyperparameters have a low impact on the efficiency in solving the binary classification problem. In addition, by comparing the results obtained at this stage with those of the classical threshold method, it can be confirmed that neural networks allow for greater classification accuracy by more than 9%. It should also be emphasized that the obtained neural network efficiency results were obtained for both scenarios, S1 and S2. However, for other measurement scenarios, the efficiency of the network and the threshold method could differ.

#### 4.3.2. Deep Learning Model Accuracy

Having obtained information about the architecture, i.e., 4 hidden layers and 256 nodes, it was possible to further analyze additional neural network efficiency metrics, as in the case of the threshold method. The average values of sensitivity and precision for both scenarios exceeded 99%. However, for the S1 scenario, the value for the convolutional network was 98.7%. These values prove that neural networks are much more sensitive and precise than the threshold method.

It was also noted that the minimum classification accuracy for individual angles was 93% and it was achieved by the convolutional network for the S1 scenario. Comparing the results for the threshold method, the decrease in accuracy for 90° and 270° angles was again obtained. However, the accuracy for these angles is 36% to 53% higher.

#### 4.3.3. Effect of Using Power of Received Signals in Input Layer

The issues raised in the cited studies related to providing various data to the input layers of neural networks and their impact on the efficiency have also been checked. It was concluded that, in addition to the total impulse response of the UWB channel, the influence of the received signal power $P_{rx}$ and $P_{fp}$ as input data on the efficiency of the artificial neural network was also checked. This approach forced the exclusive use of a multilayer perceptron as the use of convolutional layers is only justified for larger input data matrices [47]. It should be emphasized that, similarly to the previous case, the power values were normalized. However, these were the same data on the basis of which the effectiveness of the threshold method was assessed. In Table 2, the accuracy, sensitivity and precision results for both scenarios S1 and S2 are presented.

For each of the performance metrics (accuracy, sensitivity and precision), the neural network models achieved average values of 99.9%. This is further confirmation of the greater efficiency of artificial neural networks in relation to the classic threshold algorithm for the tested measurement scenarios. However, regardless of the network input data used, its efficiency increased slightly, by about 0.5%. Therefore, for the binary classification of LoS and NLoS conditions in the tested measurement scenarios, the use of the impulse response of the radio channel yields similar efficiency in the network’s operation compared to the use of the power of the received signals.

### 4.4. Antenna Orientation Angle Classification Accuracy

**Figure 9:**
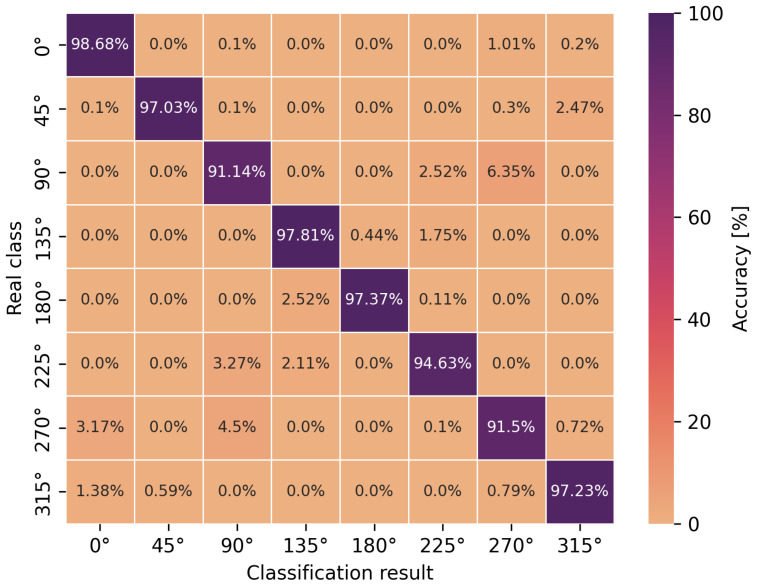
Angle classification confusion matrix for S1 scenario using a multilayer perceptron.

**Figure 10:**
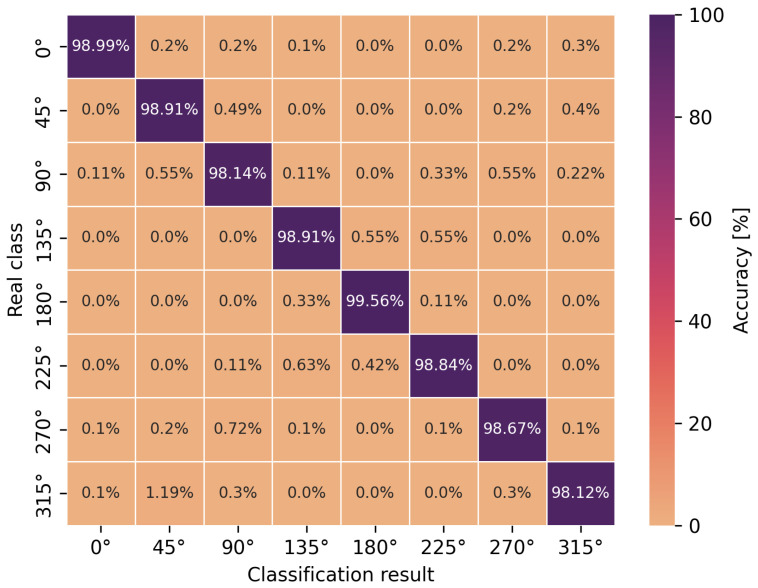
Angle classification confusion matrix for S1 scenario using a convolutional network.

**Figure 11:**
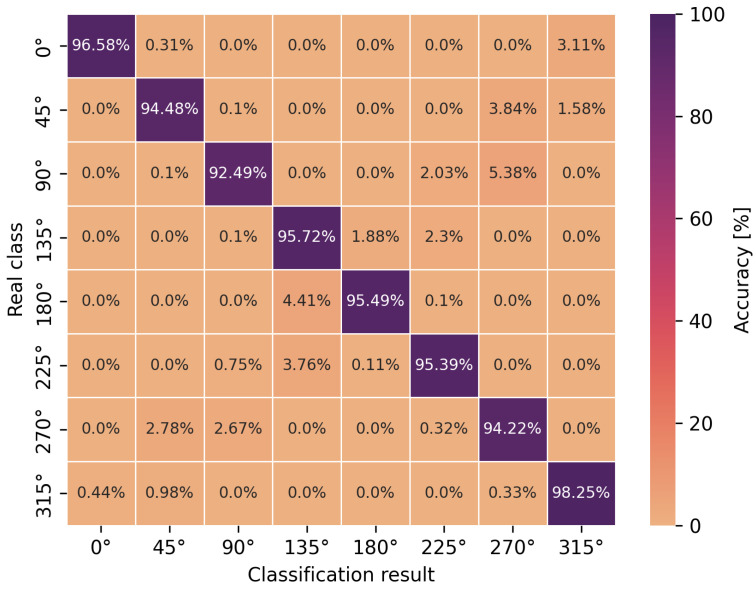
Angle classification confusion matrix for S2 scenario using a multilayer perceptron.

**Figure 12:**
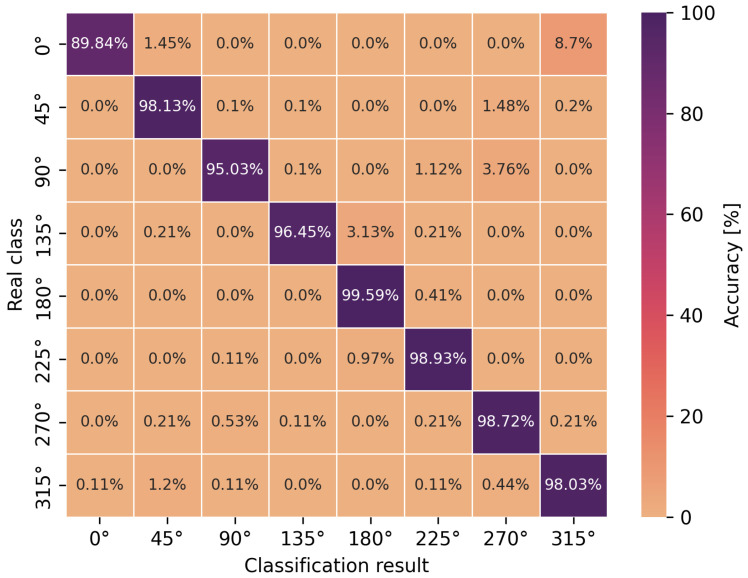
Angle classification confusion matrix for S2 scenario using a convolutional network.

The accuracy of the obtained neural network efficiency results for the classification of eight user orientation angles was determined by a confusion matrix for two types of neural networks and scenarios. It is worth emphasizing at this point that no studies have been found in the scientific literature addressing the classification of orientation angles using UWB and artificial intelligence methods, i.e., concerning position detection in relation to the antenna geometry in UWB wireless body area networks. In Figure 9, Figure 10, Figure 11 and Figure 12, the confusion matrices with accuracy values for individual angles are shown.

Based on the presented confusion matrices, it can be concluded that the overall classification accuracy is lower by 0.5–4.5% on average compared to the accuracy of binary classification networks. On the basis of the same training sets, consisting of CIRs, the connection weights of artificial neurons had to be adjusted to solve a more complicated, non-linear problem, which was the classification of multiple classes. However, it is possible to estimate the orientation angle of the antenna, defined as one of the eight angles but with less accuracy, in comparison to LoS and NLoS identification.

For the multilayer perceptron (Figure 9 and Figure 11), the accuracy of the angle classification was below 95% for the angles of 45°, 90°, 225° and 270°. Regardless of the measurement scenario, these are the same user orientation angles that contributed the most to the reduction in accuracy. In addition, it is possible to read the accuracy of the incorrect angle predictions with an error matrix. For the S1 scenario, among 8.5% of the incorrect estimations of the multilayer perceptron model for the angle of 270°, as many as 4.5% were classified as 90° and 3.17% as 0°. A similar situation occurred for the incorrect predictions of the actual angle of 90° because, among 8.86% of the errors, as many as 6.35% of the model’s indications concerned the angle of 270°. In the S2 scenario, for the multilayer perceptron, the situation was similar to the S1 scenario in terms of angle mispredictions. Thus, the confusion matrices confirm the earlier considerations regarding binary classification that the angles of 90° and 270° play a key role in the accuracy of the methods. It was also confirmed that the angles in the vicinity of the previously mentioned ones do not increase the error of the classifiers. This is due to the characteristics of the power delay profile, which differ significantly for adjacent orientation angles but are similar in shape to the curves and amplitudes for the angles symmetrical to the center of the circle.

In the case of the eight-angle classification problem, the accuracy results are improved by convolutional networks (Figure 10 and Figure 12). In general, the percentage accuracy advantage of convolutional networks over the multilayer perceptron is 3.1% for the S1 scenario and 1.5% for the S2 scenario. The greatest improvement in the classification efficiency is noticeable for the described angles of 90° and 270°, i.e., for the S1 scenario, convolutional networks achieve 7% greater accuracy compared to the multilayer perceptron. Therefore, for the more complex, non-linear problem of classifying eight antenna orientation angles, it is more efficient to use convolutional networks.

### 4.5. Antenna Orientation Angle Accuracy

**Figure 13:**
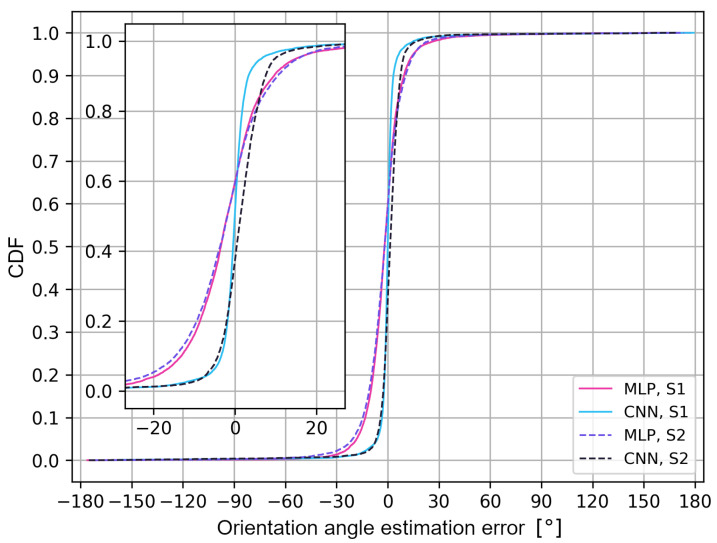
Cumulative probability of an error determining the value of the user’s orientation angle.

The last stage of the research focused on checking the effectiveness of neural network models in determining the continuous value of the antenna orientation angle. The error in the user’s orientation angle was calculated as the difference between the expected value of the orientation angle θ, i.e., the value from the set {0°, 45°, 90°, 135°, 180°, 225°, 270°, 315°}, and the value estimated by the deep neural network, $\theta^{\prime }$. The positive values of the orientation angle errors indicate the angle error that corresponds to the direction of the user’s current orientation angle, i.e., clockwise. Thus, the user orientation error values are positive from θ to 180° and negative from θ to −180°. An error greater than 180° means that it is negative, i.e., from θ to −180°. The evaluation of the models’ effectiveness for the selected architecture (4 hidden layers, 256 nodes) for various scenarios was carried out based on the error distribution function in order to determine the value of the user’s orientation angle, as shown in Figure 13.

Analyzing the results, a similar pattern in the error growth for the two types of networks and scenarios can be noticed. All the network models achieved an absolute angle error of up to 45° for 98% of the cases. The limit values of the angle estimation error were −177° and 176° (S1) and −179° and 162° (S2) for the multilayer perceptron, and −173° and 173° (S1) and −169° and 175° (S2) for the convolutional network. The greatest difference in efficiency occurs for convolutional networks, where the positive error values of the user’s orientation angle are determined between the S1 and S2 scenarios. The convolutional neural network for S1 is characterized by the highest steepness of the distribution function in the range of +/−5° of the user’s orientation angle error, thus achieving the absolute error value of the user’s orientation angle of up to 4° for 85% of the cases.

Analyzing the distribution function, it can be concluded that the error range of +/−20° for the multilayer perceptron contains 93% of the values for the S1 scenario and 98% for S2; for the convolutional networks, it has 92% of the values for the S1 scenario and 97% for S2. For the multilayer perceptron in both scenarios and for the convolutional network in the S1 scenario, negative values of the user’s orientation angle estimation error are achieved in 61% of the cases. For the convolutional network in the S2 scenario, negative values of the user’s orientation angle estimation errors are achieved in 40% of the cases. The advantage of convolutional networks over classic multilayer perceptrons in the two scenarios is also noticeable. However, the difference in the efficiency of the two types of networks is more significant for the S2 scenario.

**Figure 14:**
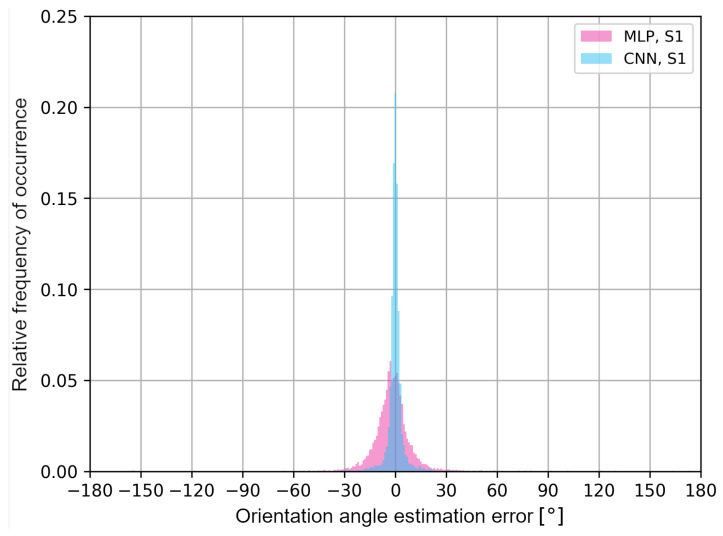
User’s orientation angle estimation error histograms for S1 scenario.

**Figure 15:**
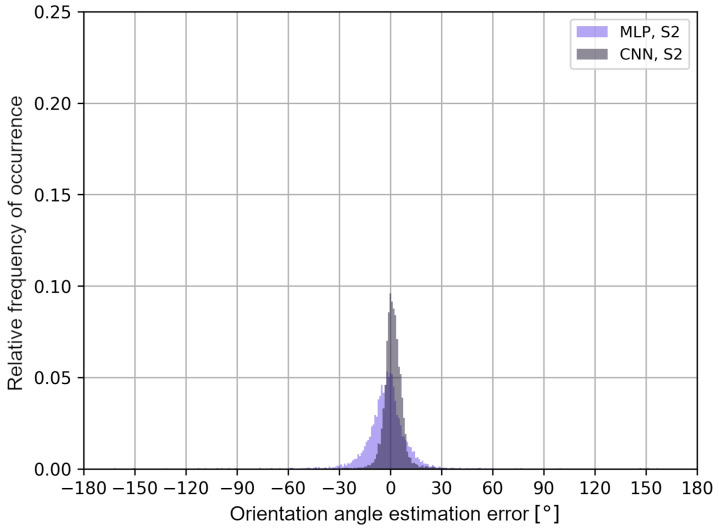
User’s orientation angle estimation error histograms for S2 scenario.

The obtained results may differ depending on the architecture of the neural networks and the analyzed measurement scenarios, which is a limitation of the presented approach. To better illustrate the distribution of the user’s orientation angle errors, in Figure 14 and Figure 15, error histograms for the neural networks tested in both measurement scenarios are presented.

The histograms in Figure 14 and Figure 15 confirm that, for multilayer perceptrons, the distribution of errors is similar in both scenarios and it is characterized by a higher frequency of negative error values of the user’s orientation angle. In the S2 scenario, the convolutional neural network shows a higher frequency of positive error values in the estimation of the user’s orientation angle. In addition, in Table 3, the average values of the classification accuracy of one of the eight angles and the maximum absolute values of the orientation angle estimation errors for a given cumulative value of the distribution function are presented.

The accuracy values in Table 3 come from the analysis in Section 4.4. Each presented classification accuracy value was taken as the cumulative probability value for regression models, and, on this basis, the maximum values of the orientation angle error were determined. As a result, much greater accuracy in determining the position angle was achieved compared to the results of the studies carried out on the subject in question described in [11]. According to the discussed results, none of the networks exceeded the value of 45°, i.e., the value of the angle between the distinguishable classes for the multi-angle classification problem. It can therefore be concluded that the angle value from the continuous interval obtained at the network output is the most detailed information in the application of orientation angle detection, ensuring the same level of information reliability. Thus, for the discussed regression problem, convolutional neural networks are more efficient than a multilayer perceptron.

## 5. Conclusions

In this paper, the UWB technique was analyzed in terms of its capability to provide additional information about the user’s location in the form of orientation relative to the antenna geometry. UWB is considered the most promising technology for accurate positioning, required in various commercial applications, such as IoT, sports bands, augmented reality, marine communication, unmanned robots [34] and car security systems, or for increased location accuracy with UWB radio modules in mobile phones and WiFi access points [51].

In order to measure the impulse response of a UWB radio channel and the power of the received signals, a pair of EVB1000 devices with DW1000 radio modules was used. The variability of the signal power and impulse response values was demonstrated in several measurement scenarios and by testing the angle of the user’s position. After carrying out the measurement campaign in two static scenarios (in the corridor and in the hall of a building), the classical threshold method and artificial intelligence methods were implemented, and the results obtained for the classification and regression problem were analyzed. Based on the results, it was proven that the threshold method for the analyzed scenarios showed a maximum of 90% accuracy for the binary classification of the user’s orientation (LoS or NLoS) for a 5 dB threshold. In the context of neural networks, it was shown that the influence of the model architecture and the type of network (the multilayer perceptron and the convolutional network) on the accuracy of binary classification was marginal in the considered scenarios. For the tested range of hidden layers and nodes, over 99% accuracy was achieved.

Then, the effectiveness of the examined structures of neural networks was checked using the classification of eight angles. The regression of determining the value of the angle from the range of 0–360°, which is impossible to perform with the classical threshold method, was studied as well. On this basis, it was proven that the accuracy of the multiclass classifier was lower by 0.5–4.5% compared to the binary classifier. However, it provides four times more accurate information about the user’s location. Finally, with the conducted research, it was confirmed that the effective use of the impulse response of the radio channel for the regression problem of determining the user’s position angle is possible. Convolutional neural networks for both tested scenarios proved more effective, achieving an angle determination error of up to 4° for about 85% of the cases.

It should be emphasized that this research does not fully cover the full scope of the problem of user orientation detection, particularly with the UWB technique. In the future, one could carry out research for different dynamic scenarios, where the influence of fast fading is anticipated to influence the effectiveness of the proposed approach [3]. The range of potential study issues includes taking into account people with different body builds [4], performing channel impulse response sampling in pre-processing before delivering the data to the input of the neural network [39], placing different additional obstacles and objects between [21] and around the user and also studying the impact of other types of neural networks and hyperparameter values on user orientation detection in relation to the antenna geometry [46].

## Figures and Tables

**Table 1:** Classification results of the threshold method proposed by Decawave.

S1 Scenario			
**NLoS Angle Range [°]**	**Accuracy [%]**	**Sensitivity [%]**	**Precision [%]**
90–270	89.12	97.47	79.1
135–225	85.52	76.62	99.94
**S2 Scenario**			
**NLoS Angle Range [°]**	**Accuracy [%]**	**Sensitivity [%]**	**Precision [%]**
90–270	87.46	100	75.54
135–225	89.88	83.54	99.97

**Table 2:** Efficiency of a multilayer perceptron using the signal power for the classification of LoS and NLoS conditions.

S1 Scenario			
**Model**	**Accuracy [%]**	**Sensitivity [%]**	**Precision [%]**
1	99.93	99.97	99.87
2	99.96	99.97	99.93
3	99.99	99.97	100
4	99.87	99.97	99.7
5	99.99	99.97	100
Mean	99.95	99.97	99.9
**S2 Scenario**			
**Model**	**Accuracy [%]**	**Sensitivity [%]**	**Precision [%]**
1	99.99	100	99.97
2	99.96	100	99.9
3	99.97	100	99.93
4	99.97	100	99.93
5	99.97	100	99.93
Mean	99.97	100	99.93

**Table 3:** Maximum error for given values of cumulative probabilities of estimated neural network responses.

Scenario	Neural Network	Avg. Classification Acc.	Max. Error [°]
S1	MLP	95.7%	⩽25
S1	CNN	98.8%	⩽37
S2	MLP	95.3%	⩽26
S2	CNN	96.8%	⩽18

## Data Availability

The datasets used in this research are available for free from the authors with the CC BY license.
